# Exome Sequencing of 18 Chinese Families with Congenital Cataracts: A New Sight of the *NHS* Gene

**DOI:** 10.1371/journal.pone.0100455

**Published:** 2014-06-26

**Authors:** Wenmin Sun, Xueshan Xiao, Shiqiang Li, Xiangming Guo, Qingjiong Zhang

**Affiliations:** State Key Laboratory of Ophthalmology, Zhongshan Ophthalmic Center, Sun Yat-sen University, Guangzhou, China; Purdue University, United States of America

## Abstract

**Purpose:**

The aim of this study was to investigate the mutation spectrum and frequency of 34 known genes in 18 Chinese families with congenital cataracts.

**Methods:**

Genomic DNA and clinical data was collected from 18 families with congenital cataracts. Variations in 34 cataract-associated genes were screened by whole exome sequencing and then validated by Sanger sequencing.

**Results:**

Eleven candidate variants in seven of the 34 genes were detected by exome sequencing and then confirmed by Sanger sequencing, including two variants predicted to be benign and the other pathogenic mutations. The nine mutations were present in 9 of the 18 (50%) families with congenital cataracts. Of the four families with mutations in the X-linked *NHS* gene, no other abnormalities were recorded except for cataract, in which a pseudo-dominant inheritance form was suggested, as female carriers also had different forms of cataracts.

**Conclusion:**

This study expands the mutation spectrum and frequency of genes responsible for congenital cataract. Mutation in *NHS* is a common cause of nonsyndromic congenital cataract with pseudo-autosomal dominant inheritance. Combined with our previous studies, a genetic basis could be identified in 67.6% of families with congenital cataracts in our case series, in which mutations in genes encoding crystallins, genes encoding connexins, and *NHS* are responsible for 29.4%, 14.7%, and 11.8% of families, respectively. Our results suggest that mutations in *NHS* are the common cause of congenital cataract, both syndromic and nonsyndromic.

## Introduction

Congenital cataract is the most common cause of childhood blindness. Its prevalence is 1–6 per 10000 live births, but it can be as high as 5–15 per 10,000 in less developed areas of the world [1]–[3]. Congenital cataracts can occur independently or be accompanied by other ocular and/or systemic abnormalities, and are thus designated as “nonsyndromic” or “syndromic” forms [4]. Genetic defect is a common cause of congenital cataract and was estimated to account for about 25% of patients [5]. Autosomal dominant inheritance is the most common form, although other forms of inheritance have also been described. To date, at least 34 genes have been reported to cause congenital cataracts (Cat-Map; http://cat-map.wustl.edu/) [6] (Table 1). Identification of the genetic basis is a great challenge in congenital cataracts, as it is highly heterogeneous in term of genetic and clinical phenotypes. Some of the known genes were selected for analysis in a cohort of patients in few studies and the results showed that the mutation frequencies have great differences [7]–[12]. We previously conducted similar studies and were able to detect mutation in 14 of 34 (41%) families with congenital cataracts [13]–[15]. In order to identify the genetic cause of the remaining and newly recruited families with congenital cataracts, whole exome sequencing was used in this study to screen the mutations, and then the detected variants were confirmed by Sanger sequencing. Nine mutations were identified in the 18 families; of those *NHS* mutations were found in four families.

**Table 1:** Information of the 34 genes referred in this study.

Gene	Inheritance	Genomic DNA	mRNA	Protein
EPHA2	AD/AR	NC_000001.10	NM_004431.3	NP_004422.2
GJA8	AD/AR	NC_000001.10	NM_005267.4	NP_005258.2
CRYGC	AD	NC_000002.11	NM_020989.3	NP_066269.1
CRYGD	AD	NC_000002.11	NM_006891.3	NP_008822.2
FYCO1	AR	NC_000003.11	NM_024513.3	NP_078789.2
BFSP2	AD/AR	NC_000003.11	NM_003571.2	NP_003562.1
CRYGS	AD	NC_000003.11	NM_017541.2	NP_060011.1
GCNT2	AR	NC_000006.11	NM_001491.2	NP_001482.1
GALT	AR	NC_000009.11	NM_000155.3	NP_000146.2
TDRD7	AR	NC_000009.11	NM_014290.2	NP_055105.2
VIM	AD	NC_000010.10	NM_003380.3	NP_003371.2
SLC16A12	AD	NC_000010.10	NM_213606.3	NP_998771.3
PITX3	AD/AR	NC_000010.10	NM_005029.3	NP_005020.1
CRYAB	AD/AR	NC_000011.9	NM_001885.1	NP_001876.1
MIP	AD	NC_000012.11	NM_012064.3	NP_036196.1
GJA3	AD	NC_000013.10	NM_021954.3	NP_068773.2
TMEM114	AD	NC_000016.9	NM_001146336.1	NP_001139808.1
HSF4	AD/AR	NC_000016.9	NM_001040667.2	NP_001035757.1
MAF	AD	NC_000016.9	NM_005360.4	NP_005351.2
CRYBA1	AD	NC_000017.10	NM_005208.4	NP_005199.2
GALK1	AR	NC_000017.10	NM_000154.1	NP_000145.1
FTL	AD	NC_000019.9	NM_000146.3	NP_000137.2
LIM2	AR	NC_000019.9	NM_030657.3	NP_085915.2
BFSP1	AR	NC_000020.10	NM_001278607.1	NP_001265536.1
CHMP4B	AD	NC_000020.10	NM_176812.4	NP_789782.1
CRYAA	AD/AR	NC_000021.8	NM_000394.2	NP_000385.1
CRYBB2	AD	NC_000022.10	NM_000496.2	NP_000487.1
CRYBB3	AD/AR	NC_000022.10	NM_000496.2	NP_000487.1
CRYBB1	AD/AR	NC_000022.10	NM_001887.3	NP_001878.1
CRYBA4	AD	NC_000022.10	NM_001886.2	NP_001877.1
NHS	XL	NC_000023.10	NM_198270.2	NP_938011.1
AGK	AR	NC_000007.13	NM_018238.3	NP_060708.1
EYA1	AD	NC_000008.10	NM_000503.4	NP_000494.2
FOXE3	AD/AR	NC_000001.10	NM_012186.2	NP_036318.1

Note: AD = autosomal dominant; AR = autosomal recessive; XL =  X-linked.

## Methods

### Patients

Written informed consent conforming to the tenets of the Declaration of Helsinki and following the Guidance of Sample Collection of Human Genetic Diseases (863-plan) by the Ministry of Public Health of China were obtained from all participating individuals or their guardians of the 18 families enrolled in this study. This study was approved by the Institutional Review Board of the Zhongshan Ophthalmic Center. Of the 18 families, ten were from our previous studies with no mutations identified [13]–[15], while the other eight families had not been analyzed before. Thirteen families with family history showed autosomal dominant inheritance, two were sporadic patients, while the other three were not clear if they had family histories. None of them were recorded to have systemic abnormalities. Ocular examinations were performed by ophthalmologists in Zhongshan Ophthalmic Center. Congenital cataract means that cataracts were noticed at birth or in the first few months. Microcornea was defined as a cornea whose horizontal diameter was less than 10 mm. The procedures for obtaining written informed consent and for preparing of genomic DNA were the same as previously described [13],[14],[16].

### Exome Sequencing

Exome sequencing was performed by Macrogen (http://www.macrogen.com/), a commercial service. The criteria to select samples for exome sequencing included: 1. The total amount of genome DNA can't be less than 5 ug; 2. There was no smear by running on an agarose gel. All the 18 samples of probands from 18 unrelated families were involved in exome sequencing. Exome capture was carried out using an Illumina TruSeq Exome Enrichment Kit (62 M) array. The kit included 340,427 probes (95 mer DNA probes) and could enrich about 201,121 exons and cover about 97.2% CCDS region. Exome-enriched DNA fragments were sequenced by an Illumina HiSeq2000; the average sequencing depth was 125-fold. Over 99% base call accuracy was up to Q20, which means that the probability of an incorrect base call is 0.01. After the low quality reads were filtered, the clean data will be aligned to the consensus sequence (UCSC hg19) to detect variants by SAMtools. Additional bioinformatics analysis of all the variants were provided from dbSNP (http://www.ncbi.nlm.nih.gov/), 1000 Genome (http://browser.1000genomes.org/index.html), PolyPhen-2 (http://genetics.bwh.harvard.edu/pph2/), SIFT (http://sift.jcvi.org/), and GERP (http://mendel.stanford.edu/SidowLab/downloads/gerp/) online tools.

### Variants Analysis

From the exome sequencing results of the 18 probands, detected variants in the 34 known causative genes were summarized. Then we excluded the variants which we don't considered pathogenic as the following criteria: 1. Minor allele frequency (MAF) ≥0.01 from 1000 Human Genome Project database; 2. Located in non-coding region without affecting splicing site; 3. Synonymous variants without affecting splicing site; 4. Only one single heterozygous variation detected in recessive genes. All the other variants were considered pathogenic and summarized for validation.

### Sanger Sequencing

Sanger sequencing was used to confirm the potential pathogenic variants detected by exome sequencing, including missense, nonsense, indels, and splice site variants. Primers used to amplify the sequence with each variant were either referred to in previous studies [13],[15],[17] or designed by online tool Primer3 (http://frodo.wi.mit.edu/primer3/) (Table S1). The nucleotide sequences of amplicons were analyzed using an ABI BigDye Terminator cycle sequencing kit v3.1 (Applied Biosystems, Foster City, CA) on an ABI 3130 Genetic Analyzer (Applied Biosystems). Sequencing results were aligned with consensus sequences from the National Center for Biotechnology Information (NCBI) human genome database (http://www.ncbi.nlm.nih.gov/), using the SeqManII program of the Lasergene package (DNAStar Inc. Madison, WI). Confirmed variants were further sequenced in the available family members and 96 unrelated control individuals. The descriptions of the variants followed the nomenclature recommended by the Human Genomic Variation Society (HGVS; http://www.hgvs.org/mutnomen/). The effects of missense variations were evaluated by the PolyPhen-2 [18] and SIFT [19] online tools, and the effects of intronic variants on splicing site changes were predicted by the Berkeley Drosophila Genome Project (BDGP; http://www.fruitfly.org/seq_tools/splice.html [20].

## Results

**Figure 1:**
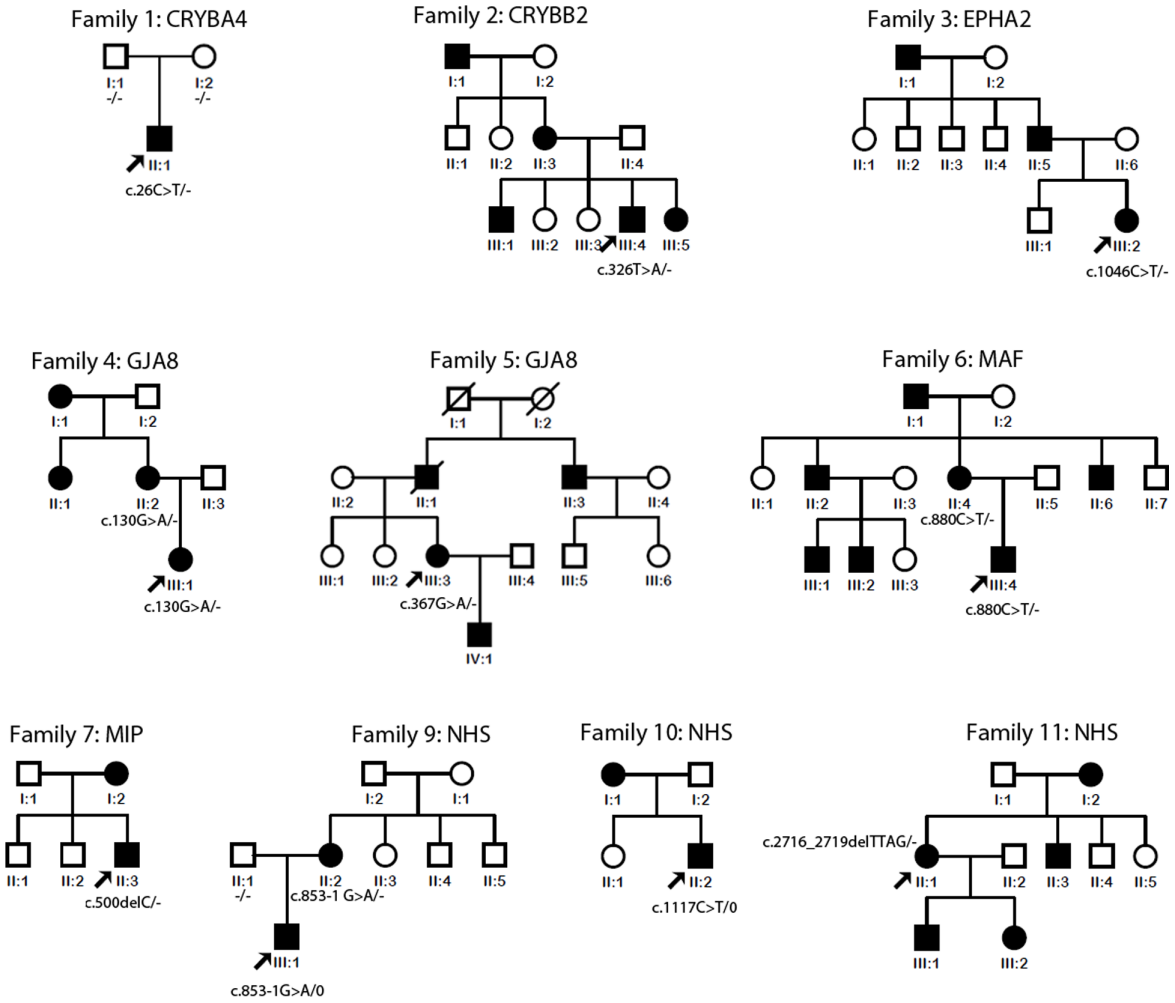
Pedigrees of the 9 available families with mutations. The family number and their causative gene were just noted above the pedigree. The mutations of available members were noted beside or below the members. The – indicated that the mutation was not present in the chromosome while the 0 indicated that males had only one X chromosome. Family 8 was recorded to have a history of congenital cataracts but the details of the pedigree are not available. The

**Figure 2:**
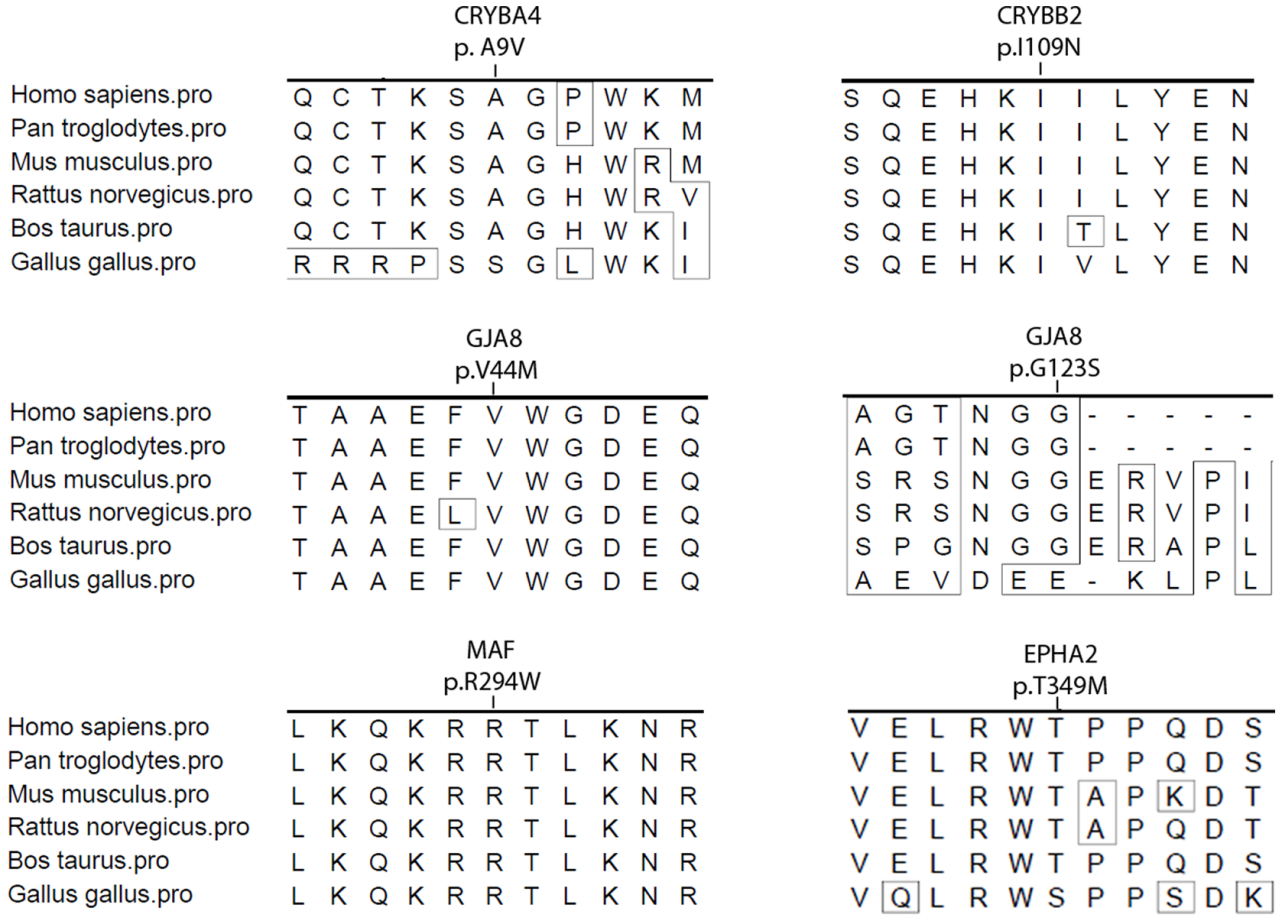
Conservation alignments for the five missense mutations. The amoni acid position with p.I109N in *CRYBB2,* p.V44M in *GJA8*, and p.R294W in *MAF* are highly conserved, while the p.A9V in *CRYBA4, the p.T349M inEPHA2, and the p. G123S in GJA8 are* not highly conserved.

From the whole exome sequencing results of the 18 probands, a total of 1545 variants were identified in the 34 known genes. Twelve variants of them were considered potential pathogenic after we excluded all the variants which were not pathogenic. And 11 of them were confirmed while one was false positive by Sanger sequencing. The 11 variants were present in seven genes and were identified in 11 of the 18 families with congenital cataracts (Table 2, Table 3, Figure 1, and Figure S1). Two of the 11 variants were predicted to be benign (Table 3), while the other 9 were likely to be pathogenic (Table 2). None of the 11 variants was found in 96 normal controls. Four of the 11 variants were identified in *NHS* in four of the 18 (22.2%) families. The other seven mutations were identified in six genes, including two mutations in *GJA8* (gap junction protein, alpha-8; MIM: 600897), and one mutation each in *CRYBA4* (crystallin, beta-A4; MIM: 123631), *CRYBB2* (crystallin, beta-B2; MIM: 123620), *EPHA2* (ephrin receptor EphA2; MIM: 176946), *MAF* (v-maf avian musculoaponeurotic fibrosarcoma oncogene homolog; MIM: 177075), and *MIP* (major intrinsic protein of lens fiber; MIM: 154050). The four mutations in *NHS* included two nonsense, one frameshift, and one splice site mutations. Analysis of available family members in three families demonstrated cosegregation of the mutation with the disease (Figure 1; families 4, 6, and 9). Conservation alignment analysis of the six missense mutations showed relatively conserved residues (Figure 2). None of the 11 variants was present in the 96 normal controls.

**Table 2:** The 9 mutations identified in 9 of 18 Chinese families with congenital cataract.

		Variations			Bioinformation prediction			Variations in		
Family ID	Gene	Nucleotide	Amino acid	Status	Polyphen-2	SIFT	Splice	patients	controls	Note
Family 2	CRYBB2	c.326T>A	p.I109N	Hetero	PD	D	/	1/18	0/96	Novel
Family 3	EPHA2	c.1046C>T	p.T349M	Hetero	PD	D	/	1/18	0/96	rs200490325
Family 4	GJA8	c.130G>A	p.V44M	Hetero	PD	D	/	1/18	0/96	Novel
Family 6	MAF	c.880C>T	p.R294W	Hetero	PD	D	/	1/18	0/96	Novel
Family 7	MIP	c.500delC	p.A169Pfs*15	Hetero	/	/	/	1/18	0/96	Novel
Family 8	NHS	c.556G>T	p.E186*	Hetero	/	/	/	1/18	0/96	Novel
Family 9	NHS	c.853-1G>A	/	Hemi	/	/	DSA	1/18	0/96	Novel
Family 10	NHS	c.1117C>T	p.R373*	Hemi	/	/	/	1/18	0/96	Reported (rs132630322)
Family 11	NHS	c.2716_2719delTTAG	p.L906Mfs*24	Hetero	/	/	/	1/18	0/96	Novel

Note: Hetero = heterozygosity; Hemi = hemizygosity; D = damaging; PD = probably damaging; DSA =  donor site abolished.

**Table 3:** The two variants that can't be excluded.

		Variations			Bioinformation prediction			Variations in		
Family ID	Gene	Nucleotide	Amino acid	Status	Polyphen-2	SIFT	Splice	patients	controls	Note
Family 1	CRYBA4	c.26C>T	p.A9V	Hetero	Benign	Tolerate	/	1/18	0/96	Novel
Family 5	GJA8	c.367G>A	p.G123S	Hetero	Benign	Tolerate	/	1/18	0/96	COSM1333689

Note: Hetero = heterozygosity.

**Figure 3:**
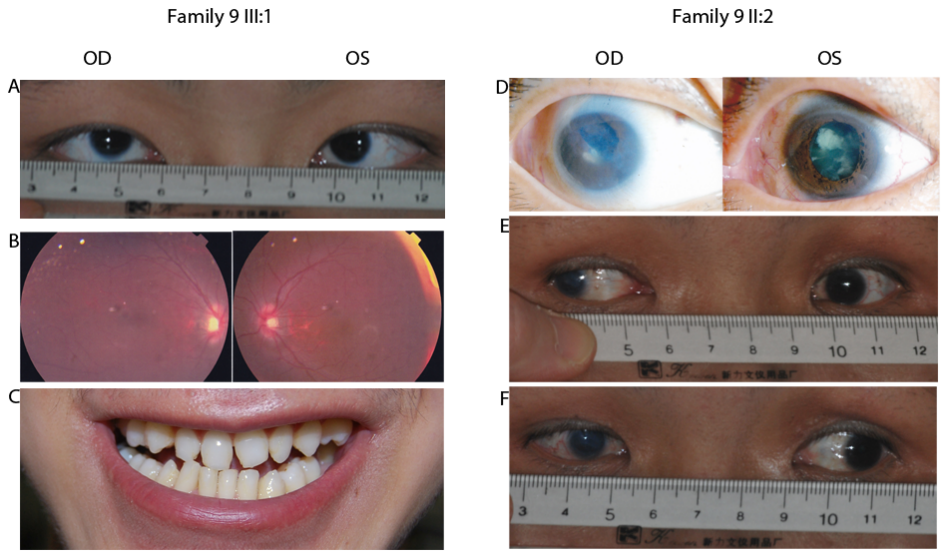
The clinical examination details of patients from the family 9. A–C showed that the member III∶1 had bilateral10 mm of the horizontal corneal diameter (A), bilateral mild tigroid retinal change in the temporal region of the optic disc (B), and dental condition without obvious screwdriver incisors or mesiodens (C). D–F showed that the member II∶2 had posterior capsule opacification in her left eye and postoperative pupil shift in her right eye (D), 10 mm of the horizontal corneal diameter in her left eye (E), and 8 mm in her right eye (F).

The clinical data of the probands with mutations are listed in Table 4. Of the four families with mutations in *NHS*, an X-linked gene, three (family 9, family 10, and family 11) showed congenital cataracts, as well as microcornea and nystagmus. The fourth one, family 8, with *NHS* c.556G>T (p.E186*) mutation, showed punctate cataract and high myopia (−7.5D for the right eye and −8.0D for the left eye). There were no records of other abnormalities in the patients with *NHS* mutations. Clinical re-examination was only possible for two affected individuals and an unaffected member (II:1, II∶2, and III∶1) of family 9, with the c.853-1G>A mutation in *NHS*. The proband (III∶1) harbored a hemizygous mutation and his affected mother (II∶2) harbored a heterozygous mutation, while his unaffected father (II∶1) did not carry the mutation. The proband (Figure 1; family 9-III∶1) had undergone cataract surgery nine years prior to the study, so that cataract was no longer observed (Figure 3A). His affected mother (Figure 1; family 9-II∶2) had posterior subcapsule opacification in her left eye; her right eye underwent cataract surgery when she was a teenager (Figure 3D). The horizontal corneal diameter of the proband (III∶1) was 10 mm in both eyes (Figure 3A) while those of his affected mother (II∶2) were 9 mm in the left eye (figure 3E) and 8 mm in the right eye (Figure 3F). Both the mother and son had bilateral nystagmus. In addition, the proband (III∶1) showed bilateral tigroid retinal change in the temporal region of the optic disc (Figure 3B). The proband (III∶1) had abnormal teeth (figure 3C), but atypical for Nance-Horan syndrome. His mother had lost all her teeth when she was forty.

**Table 4:** Clinical features of affected individuals with variants identified in this study.

	Variations		Sex	Age (yrs) at		Inheri-	Visual acuity	Cataract types	Cornea diameter	Nystagmus	Other abnormalities
Family ID	Gene	Nucleotide		Exam	Onset	tance	Right;Left		Right;Left		
Family 1	CRYBA4	c.26C>T	M	5	NA	S	0.2;0.1	Lamellar, punctate	NA	No	Myopia
Family 2	CRYBB2	c.326T>A	M	NA	NA	AD	0.07;0.1	membrane cataract	10 mm;10 mm	Yes	No
Family 3	EPHA2	c.1046C>T	F	6	FMB	AD	0.1;0.1	Nuclear	NA	No	StrabismusOS
Family 4	GJA8	c.130G>A	F	7	FMB	AD	0.1;0.1	NA	NA	Yes	No
Family 5	GJA8	c.367G>A	M	31	NA	AD	FC;0.6	Nuclear	NA	No	StrabismusOD
Family 6	MAF	c.880C>T	M	0.3	FMB	AD	NA	Nuclear	NA	Yes	No
Family 7	MIP	c.500delC	M	30	FMB	NA	0.01;0.01	Nuclear	10 mm;10 mm	Yes	No
Family 8	NHS	c.556G>T	F	5	NA	NA	0.3;0.2	Punctate	NA	No	Myopia
Family 9	NHS	c.853-1G>A	M	16	FMB	XL	0.05;0.05	NA	10 mm;10 mm	Yes	No
Family 10	NHS	c.1117C>T	M	19	FMB	XL	FC;FC	Nuclear	8 mm;8 mm	Yes	No
Family 11	NHS	c.2716_2719delTTAG	F	0.5	FMB	XL	PL;PL	Nuclear	9 mm;9 mm	Yes	No

Note: NA = not available; S = sporadic; FMB = first few month; AD = autosomal dominant; XL = X-linked; FC = finger counting; PL = pursuit of light; OD = right eye; OS = left eye.

## Discussion

In this study, we performed whole exome sequencing on probands from 18 families with congenital cataracts. Analysis of the sequencing information for 34 genes known to be associated with congenital cataract revealed 12 potential pathogenic mutations. Sanger sequencing confirmed the 11 of them and nine mutations in six genes were considered to be pathogenic in 9 (50.0%) of the 18 families.

To date, about 34 genes have been reported to be associated with congenital cataract (Cat-Map). About half of the mutations were from genes encoding crystallins and 15% of the mutations were from genes encoding connexins [21]. Some screening studies on a set of the two groups of genes in a cohort of patients showed that the frequencies were much lower than expected. Hansen et al. selected 28 Danish families with hereditary congenital cataracts to screen 17 genes and found that mutations in genes encoding crystallins and connexins accounted for 53.5% [7]. Other studies have screened only a few genes in different populations and all of their results showed that the mutation frequencies were no more than 20% [7]–[10],[12],[22]. In our previous study, we screened all of the 12 genes encoding crystallins and connexins in 25 Chinese families, and 40% of the families were found to carry mutations [13]. Regarding other genes, most reports were based on an individual gene in one family. Therefore, it is still not clear about the mutation frequencies of the known genes in a group of patients with congenital cataracts, especially it can be different in specific populations.

In the 9 potential variants identified in the current study, four were in NHS gene and were detected in four X-linked families, which we previously considered as autosomal dominant inheritance. Combining our previous studies with the current study, we identified mutations in 23 of 34 recruited families with congenital cataract, accounting for 67.6% (23/34) of the families [13]–[15]. Mutation frequencies in genes encoding crystallins, genes encoding connexins, and the *NHS* gene were 29.4% (10/34), 14.7% (5/34), and 11.8% (4/34), respectively. Our results indicated that the NHS gene is also a major causative gene besides the above two groups of genes, especially in some congenital cataracts with pseudo-autosomal dominant inheritance.

NHS gene is located in Xp22.13, and mutations in NHS can cause X-linked dominant Nance–Horan syndrome, which is also known as X-linked congenital cataract [23],[24] or X-linked cataract–dental syndrome [25]. Affected males with Nance–Horan syndrome typically show severe congenital cataracts and dental abnormalities, with occasional dysmorphic features and mental retardation, while females have milder symptoms[26]. While 100% of patients with Nance–Horan syndrome have bilateral congenital cataracts, only 65% have typical dental anomalies, including screwdriver incisors and mesiodens [26]. In some cases, Nance–Horan syndrome was diagnosed after mutations were identified in the NHS gene of affected males who were first noted to suffer from severe congenital cataracts [27]. In our study, all patients in the four families with NHS gene mutations were recruited as congenital cataract, and only three (family 9, family 10, and family 11) had microcornea and nystagmus, which are major manifestations of Nance–Horan syndrome. However, there were no records regarding abnormal dental features. Although it has been demonstrated in a study that Nance–Horan syndrome and X-linked cataract are allelic disorders, there have been no studies to date regarding mutation frequency in congenital cataracts [24]. Therefore, we strongly suggest that the NHS gene should be considered one of the major genes associated with congenital cataract.

In conclusion, combined with our previous studies [13]–[15], based on a total of 34 analyzed families, the results showed that mutations in the 34 known genes were responsible for about 67.6% of this set of Chinese families with congenital cataracts. And mutations in the NHS gene were identified in 11.8% of the families, in whom congenital cataract was the only recorded sign in their first visit. Atypical teeth abnormality was really detected in one patient, while there were no records about other abnormalities in the other three families with *NHS* mutations except for cataract. Therefore, we supposed that maybe it was not taken too much attention on the abnormal dental and face features caused by *NHS* mutations, especially in Chinese patients. We suggest that mutations in *NHS* are a common cause of congenital cataract, both syndromic and nonsyndromic.

## Supporting Information

Figure S1 **Sequence chromatography.** The family number was shown in the left column. Sequences with mutations from patients and normal controls were shown in the middle and right column, respectively. Each mutation was noted under the corresponding sequence. For the Family 9, the proband and his affected mother showed the hemizygous mutant sequence (upper one in the middle column) and the heterozygous mutant sequence (the lower one in the middle column), respectively.
(TIF)

Table S1 **Primers used to amplify and sequence the variants regions in this study.** This table listed 12 pairs of primers which were used to amplify the genomic fragments with variants detected by exome sequencing.
(XLSX)
