# Supplementary figures and images for: Exome Sequencing of 18 Chinese Families with Congenital Cataracts: A New Sight of the NHS Gene
Source: PLoS One. 2014 Jun 26;9(6):e100455. doi: 10.1371/journal.pone.0100455 (PMC4072665; doi:10.1371/journal.pone.0100455)

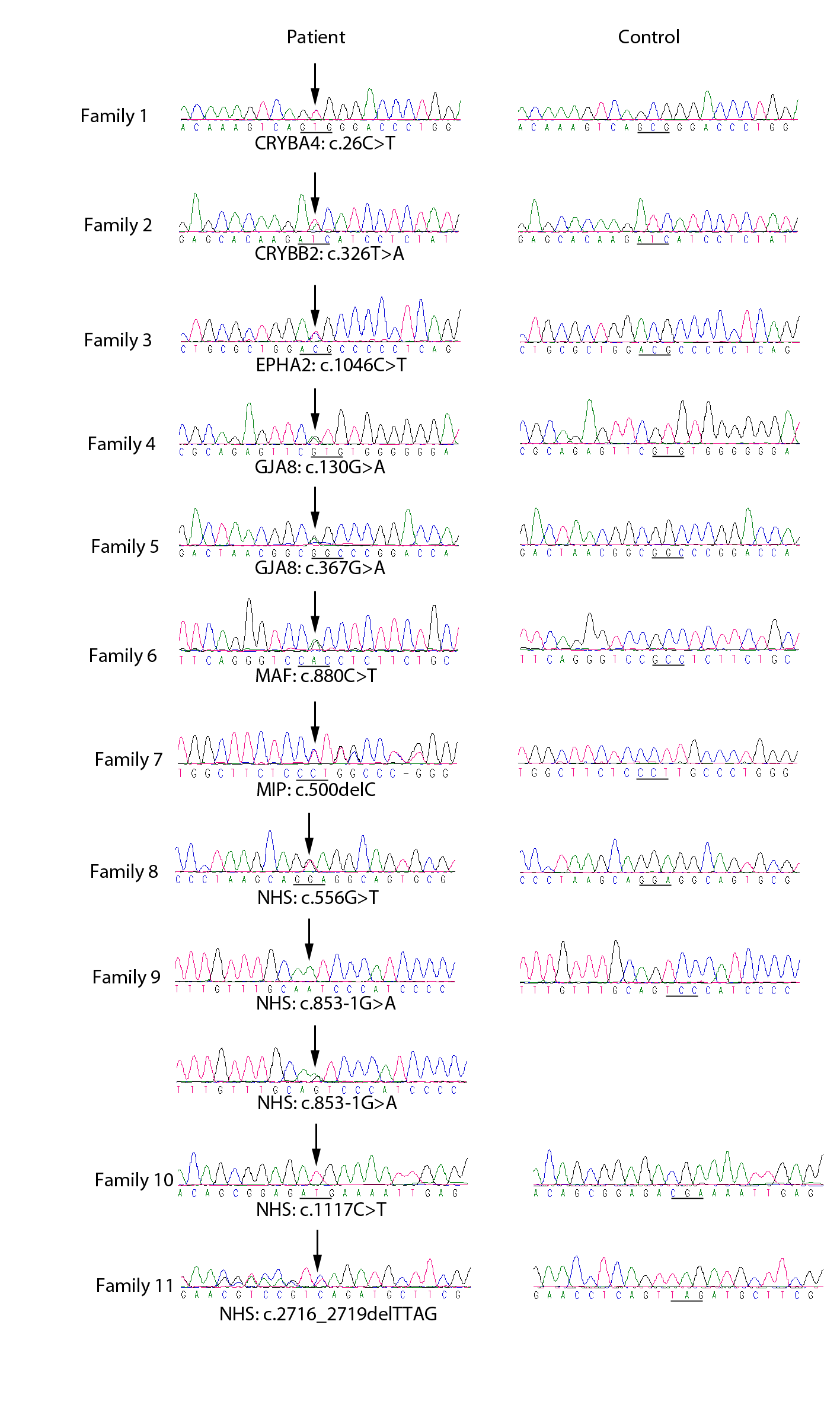

Supplement: Figure S1 — Sequence chromatography. The family number was shown in the left column. Sequences with mutations from patients and normal controls were shown in the middle and right column, respectively. Each mutation was noted under the corresponding sequence. For the Family 9, the proband and his affected mother showed the hemizygous mutant sequence (upper one in the middle column) and the heterozygous mutant sequence (the lower one in the middle column), respectively. (TIF) [file pone.0100455.s001.tif]
